# Mechanistic Insights into Wildlife Cancer and Conservation Strategies Under the One Health Framework

**DOI:** 10.3390/vetsci13080815

**Published:** 2026-08-17

**Authors:** Qiangqiang Wang, Xiaoxuan Feng, Yurun Su, Naiwen Zhang, Yevheniia Dudnyk, Hongxuan He

**Affiliations:** 1 Ministry of Education Key Laboratory for Animal Pathogens and Biosafety, College of Animal Science and Technology, Henan Institute of Science and Technology, Xinxiang 453003, China; vet_wang@126.com (Q.W.);; 2 Henan Province Livestock Genome Editing and Biobreeding Engineering Research Center, School of Life Sciences, Henan University, Kaifeng 475004, China; 3 Henan Provincial Key Laboratory of Zoonoses of Companion and Wild Animals, Henan Institute of Science and Technology, Xinxiang 453003, China; 4 Polymerase Chain Diagnostics Research Laboratory, Sumy National Agrarian University, Herasym Kondratiev 160, 40021 Sumy, Ukraine; e.dudnyk@snau.edu.ua; 5 Institute of Zoology, Chinese Academy of Sciences, Beijing 100101, China

**Keywords:** wildlife cancer, cancer ecology, environmental carcinogenesis, One Health, conservation biology

## Abstract

Cancer is increasingly recognized as an emerging concern in wildlife health and biodiversity conservation in the Anthropocene. Although traditionally viewed as an individual disease associated primarily with aging, wildlife cancer is shaped by complex interactions among environmental changes, species-specific evolutionary adaptations, and ecological processes. This review synthesizes current knowledge on the ecological and evolutionary drivers of wildlife cancer by integrating evidence from comparative oncology, environmental toxicology, wildlife pathology, and conservation biology. We examine how anthropogenic stressors, including pollution, habitat degradation, climate change, and infectious agents, influence cancer susceptibility in wild populations, and summarize intrinsic mechanisms underlying interspecific variation in cancer vulnerability, including Peto’s paradox, enhanced tumor suppression, and adaptive immune surveillance. We further highlight major methodological challenges, including limited surveillance capacity, fragmented datasets, taxonomic biases, and insufficient integration between cancer biology and conservation science. Finally, we discuss emerging interdisciplinary approaches, such as standardized monitoring frameworks, multi-omics technologies, artificial intelligence-assisted diagnosis, and One Health-based strategies, to advance wildlife cancer research and management. Collectively, this review positions wildlife cancer as an important ecological and evolutionary phenomenon and provides perspectives for incorporating cancer surveillance into biodiversity conservation and ecosystem health assessment.

## Simple Summary

Cancer is increasingly recognized as an emerging threat not only to humans and domestic animals but also to wildlife. However, the causes and consequences of cancer in wildlife remain poorly understood. This review summarizes current knowledge of how environmental changes, including pollution, habitat disturbance, and climate change, influence cancer development in wildlife. We also discuss why some species are naturally more resistant to cancer than others and how these differences have evolved. Our review highlights that wildlife cancer is closely linked to ecosystem health and can provide valuable insights into the impacts of human activities on the natural environment. We identify major challenges in wildlife cancer research, including limited surveillance and insufficient data, and propose future directions that integrate animal health research, environmental protection, and biodiversity conservation. Improving our understanding of wildlife cancer will strengthen biodiversity conservation and enhance our ability to detect environmental risks that may also affect human and animal health.

## 1. Introduction

Wild animals play essential roles in maintaining ecosystem stability through pollination, trophic regulation, and scavenging, thereby supporting ecosystem function and biodiversity. Their population dynamics and health status also serve as valuable indicators of ecosystem health. For example, amphibians, owing to their permeable skin and high metabolic rates, are particularly sensitive to water and air pollution as well as climate change, earning them the designation of “environmental sentinels” [1]. Monitoring wildlife health therefore enables the early detection of environmental degradation, including heavy metal contamination and emerging pathogens, thereby providing a critical window for timely intervention.

Despite a long evolutionary history that has endowed wild animals with remarkable adaptive capacity, wildlife health now faces unprecedented and interacting threats. Infectious agents have long posed substantial risks to wildlife populations. For example, chytridiomycosis has contributed to population declines in more than 500 amphibian species worldwide since the 1990s [2], and canine distemper virus (CDV) has caused declines of up to 90% in some African wild dog populations [3]. Human activities further intensify these threats. Habitat fragmentation increases contact among wildlife, livestock, and humans, facilitating cross-species pathogen transmission, while climate change expands the geographic range of vector-borne diseases [4]. In parallel, emerging anthropogenic threats, particularly environmental pollution and cancer, are becoming increasingly prominent, with multiple stressors interacting through interconnected One Health pathways.

Recently, wildlife cancer has been increasingly recognized as a new focus in One Health research. This review summarizes the current state of knowledge on wildlife cancer, systematically examines its ecological and biological drivers, and discusses strategies to address existing limitations in wildlife cancer surveillance, prevention, and management. By integrating perspectives from wildlife conservation, environmental governance, and public health, this review aims to advance understanding of the ecological significance of wildlife cancer within the One Health framework.

## 2. Materials and Methods

This narrative synthesis follows standard narrative review frameworks commonly adopted in wildlife comparative oncology and One Health conservation research. Literature retrieval was conducted using two core academic databases, namely Web of Science Core Collection (https://www.webofscience.com/wos/, accessed on 13 July 2026) and PubMed (https://pubmed.ncbi.nlm.nih.gov, accessed on 13 July 2026). Multiple combinations of search keywords were used for cross-database searches. Primary search terms included “wildlife cancer”, “neoplasia”, “environmental carcinogenesis”, “transmissible cancer”, “Peto’s paradox”, “comparative oncology”, “wildlife pathology”, and “biodiversity conservation”. Supplementary keywords included microplastic toxicity, climate change, tumor immune surveillance, multi-omics, artificial intelligence-assisted diagnosis, and wildlife conservation management.

Our search was not restricted by publication date, and only English-language articles were included. Two separate screening rounds were implemented to reduce selection bias. The first round screened titles and abstracts to remove irrelevant records. The full texts of the remaining papers were assessed against unified inclusion criteria in the second round. Three inclusion criteria were established. First, eligible studies were required to provide original field pathological records, population monitoring data, or controlled experimental evidence describing spontaneous or transmissible neoplasms in free-living wildlife. Second, selected studies were required to explore ecological, evolutionary, or molecular mechanisms connecting anthropogenic environmental stressors to wildlife tumorigenesis. Third, peer-reviewed empirical investigations, systematic reviews, and cohort analyses focusing on wildlife cancer risk assessment, detection, and conservation intervention were eligible for inclusion. This standardized search and screening process improves the transparency and reproducibility of this review. It also helps readers recognize taxonomic, geographic, and methodological biases within current wildlife cancer research.

## 3. The Urgency and Complexity of Wild Animal Cancer Research

**Figure 1:**
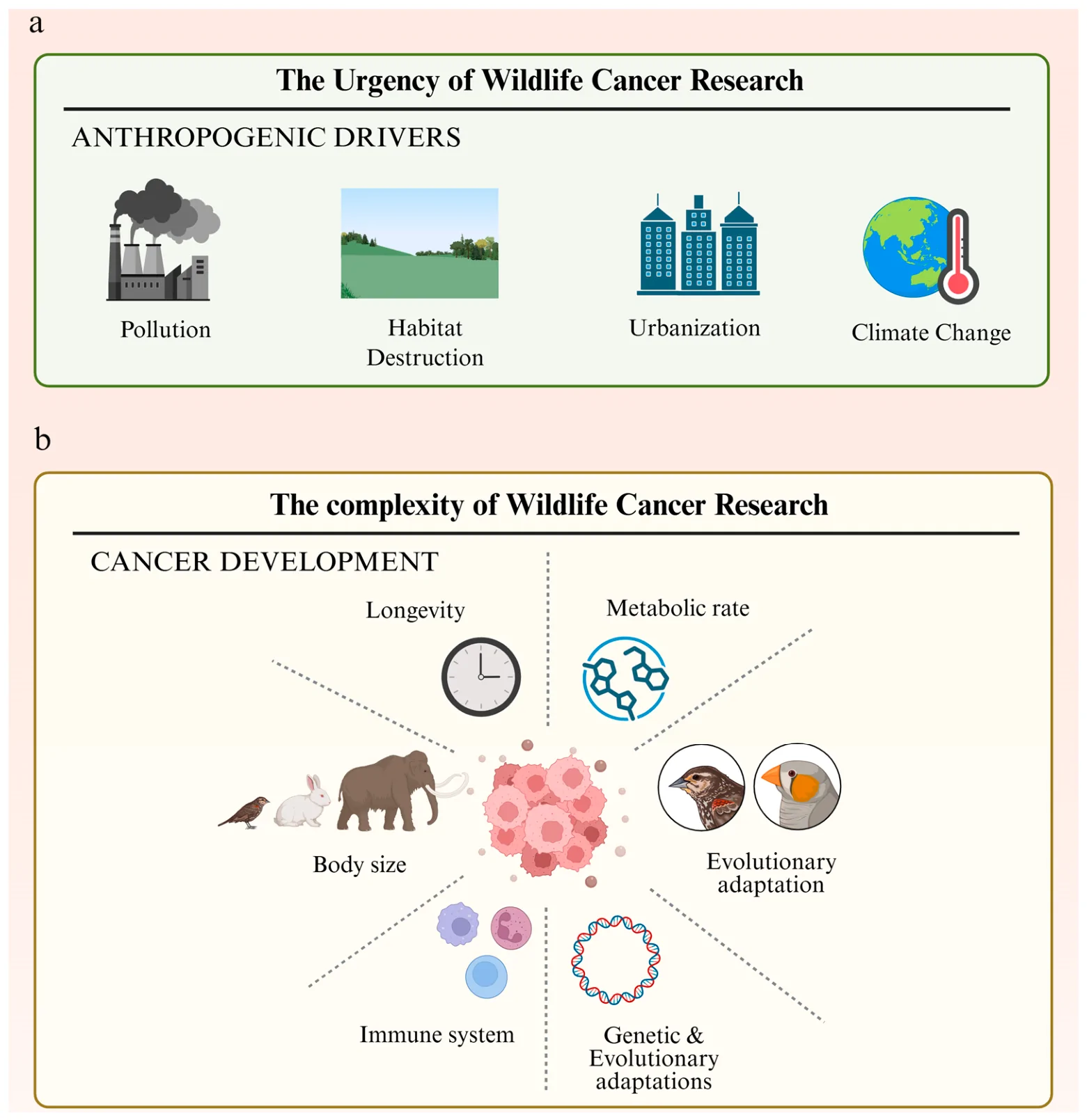
The urgency and complexity of wildlife cancer research. (**a**) Anthropogenic drivers accelerating carcinogenic risks in wild populations. (**b**) The multifaceted biological and ecological factors influencing cancer development across diverse taxa.

Wildlife cancer is increasingly recognized as an important yet historically overlooked component of wildlife health [5]. Although neoplastic diseases have been documented in numerous free-ranging vertebrate and invertebrate species, their ecological significance has long received far less attention than that of infectious diseases or habitat degradation [6]. Growing interest in wildlife cancer has emerged over recent decades, largely because accelerating anthropogenic environmental change, including pollution, habitat fragmentation, urbanization, and climate change, has substantially altered the intensity, duration, and diversity of carcinogenic exposures experienced by wild populations [5]. Consequently, cancer is now regarded not only as an individual pathological condition but also as a potential driver of reduced fitness, altered population dynamics, and biodiversity loss in susceptible species [7] (Figure 1a).

Unlike cancer research in humans or domestic animals, wildlife cancer research must account for exceptional biological and ecological heterogeneity [5]. Species differ markedly in lifespan, body size, metabolic rate, immune strategies, reproductive history, and evolutionary adaptations, all of which influence cancer susceptibility, tumour progression, and host responses [8]. Moreover, ecological processes, including migration, trophic interactions, environmental contamination, and habitat characteristics, can influence both carcinogen exposure and disease outcomes, creating complex interactions across multiple biological levels. These characteristics limit the direct extrapolation of findings from human oncology to wildlife systems and highlight the importance of comparative and evolutionary perspectives for understanding cancer across taxa [9] (Figure 1b).

Another defining characteristic of wildlife cancer research is its inherently interdisciplinary nature [10]. In natural populations, cancer represents not only a molecular process but also an ecological, evolutionary, and conservation phenomenon. Understanding its occurrence therefore requires the integration of diverse lines of evidence, ranging from pathology and genomics to behavioural ecology and environmental sciences. Furthermore, unlike clinical oncology, where diagnosis and treatment focus primarily on individual patients, wildlife cancer research aims to understand disease processes at the population and ecosystem levels, emphasizing ecological consequences rather than therapeutic intervention [11]. Consequently, wildlife cancer research has evolved into a highly interdisciplinary field, integrating comparative oncology, ecology, evolutionary biology, veterinary pathology, and conservation science. This integration provides opportunities to investigate how environmental change shapes cancer evolution across species [12].

## 4. Pollution-Mediated Mechanisms of Carcinogenesis in Wild Animals

**Figure 2:**
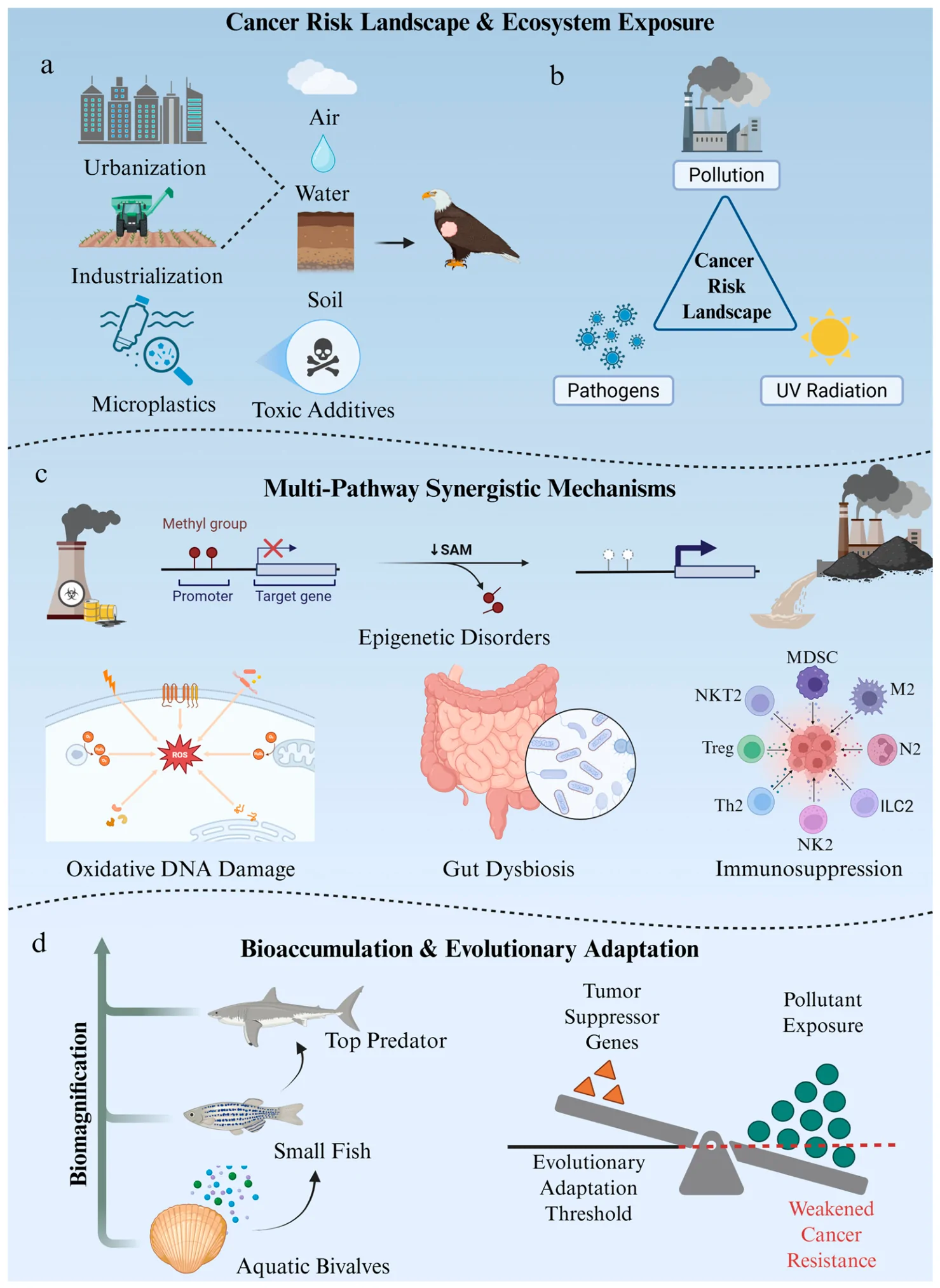
Pollution-mediated mechanisms of carcinogenesis and ecosystem exposure in wild animals. (**a**) Exposure pathways of anthropogenic pollutants via air, water, and soil into wild animal habitats. (**b**) The “cancer risk landscape” framework in wildlife. (**c**) Multi-pathway synergistic mechanisms of pollutant-driven tumorigenesis. (**d**) Ecological and evolutionary consequences of pollution.

Anthropogenic pollution has led to rapidly increasing concentrations of carcinogens (e.g., industrial emissions, plastic particles, polycyclic aromatic hydrocarbons (PAHs)) in wildlife habitats [13], thereby substantially increasing the risk of carcinogen exposure in wild populations [14]. Urbanization and industrialization further elevate exposure through multiple pathways [15]. For example, migratory species encounter pollutants via air, water, and soil [16]. Emerging contaminants, such as microplastics and nanoplastics, further increase exposure risk by causing physical tissue damage, releasing chemical additives, and acting as carriers of carcinogens [13,17]. Climate change (e.g., warmer temperatures enhance the biological activity of pollutants) and habitat degradation (which forces animals into highly contaminated environments) act synergistically to amplify carcinogenic effects [18] (Figure 2a). Pollution has been clearly categorized as an independent cancer-causing agent and together with ultraviolet radiation and pathogens, constitutes the “cancer risk landscape” [19]. Epidemiologic evidence further demonstrates that air pollutants significantly increase cancer incidence and mortality [20] (Figure 2b).

Pollutants drive tumorigenesis through multiple interacting mechanisms. Air pollutants can induce aberrant DNA methylation and histone modifications, leading to epigenetic dysregulation in human and experimental systems [21]. Absorbed organic pollutants undergo phase-one biotransformation in wildlife hepatic tissues to generate electrophilic reactive intermediates [22]. These chemical metabolites covalently bind to DNA bases and histone proteins, inducing persistent chromatin remodeling and abnormal gene transcription during cell proliferation [23]. This epigenetic alteration pathway should be regarded as an extrapolated inference from human or laboratory model systems because validation through targeted epigenetic sequencing remains limited across wild vertebrate populations [24]. Chemical pollutants also activate metabolic detoxification pathways, leading to the accumulation of reactive oxygen species (ROS), oxidative DNA damage, cell-cycle dysregulation [25], and metabolic abnormalities such as hepatic steatosis [26]. Excess reactive oxygen species induce DNA strand breaks and base mismatches during genome replication [27]. Unrepaired genetic lesions can accumulate during repeated cell division [28]. This accumulation may disrupt cell-cycle checkpoints that normally prevent the proliferation of damaged cells [29]. The oxidative damage cascade is partially supported by field-derived evidence from wildlife, including industrial harbor fish and coastal marine mammal populations [30]. Alterations in the gut microbiota may also contribute to colorectal carcinogenesis through pathways such as the “adenoma-cancer sequence” and the “jagged pathway” [31], although this mechanism has not been directly established in wildlife. In addition, pollutants impair immune surveillance and enhance the tumorigenic effects of oncogenic pathogens, particularly in aquatic organisms [25]. Beyond persistent inflammation, accumulating evidence indicates that chronic infections promote tumor development through multiple complementary mechanisms [32]. Continuous inflammatory stimulation disrupts epithelial barrier integrity and creates a microenvironment favorable for malignant transformation [33]. Persistent immune dysregulation weakens immune surveillance and facilitates the survival of transformed cells [34]. In addition, infection-associated signaling may induce epithelial-to-mesenchymal transition, thereby enhancing tumor invasion and metastatic potential in experimental systems [35]. However, direct validation of these molecular pathways in wild populations remains uneven. Collectively, these mechanisms provide a broader framework for understanding how infectious agents contribute to cancer development in wildlife. They also highlight potential directions for future comparative oncology research. Although pollutant mixtures are thought to promote cancer through multifactorial synergistic effects, the mechanisms underlying long-term, low-dose exposure remain poorly understood [14] (Figure 2c).

Ecological processes also play an important role in pollution-associated wildlife tumorigenesis. Contaminants are enriched through the food chain, concentrating carcinogens cascading from aquatic bivalves to top predators, creating a cross-species health crisis. Persistent organic pollutants accumulate in the blubber of coastal California sea lions and may increase carcinoma risk in combination with oncogenic herpesvirus infection and immune dysfunction [36]. Similarly, benthic fish from industrial harbor zones exhibit elevated rates of hepatic neoplasia associated with polycyclic aromatic hydrocarbon exposure, providing evidence that pollution-related cancers can serve as sentinel indicators of aquatic ecosystem degradation [37]. Green sea turtle fibropapillomatosis provides a representative example linking anthropogenic coastal pollution, tumor-associated viral infection, and population fitness [38]. Chelonid alphaherpesvirus 5 infection can contribute to tumor development under conditions of environmental stress, while heavy metals and polycyclic aromatic hydrocarbons may promote tumor progression through oxidative stress and immunosuppression [39]. Fibropapillomatosis can impair locomotion, feeding, and predator avoidance, thereby reducing juvenile survival and potentially constraining reproductive output in affected populations [40]. Urbanization-related stressors, including light pollution, noise pollution, and plastic waste, may also indirectly increase cancer risk by altering animal behavior and diet [41]. Consequently, tumor occurrence in wildlife may provide a sensitive indicator of ecosystem health [14]. Migratory species are key indicator species due to their wide ranges [14]. Although some populations are able to resist cancer through evolutionary mechanisms such as tumor suppressor genes [42], their ability to resist cancer may be weakened when the rate of pollutant exposure exceeds the threshold of evolutionary adaptation [43] (Figure 2d).

Overall, pollution drives wildlife cancer development through multiple mechanisms and pathways and is highly correlated with human activities. Future research needs to combine cross-species monitoring, mixed-pollutant exposure experiments [14], and evolutionary medicine frameworks to analyze the common mechanisms of pollution carcinogenesis and provide a basis for wildlife conservation and “One Health” strategies [5].

## 5. Species-Intrinsic Mechanisms Underlying Cancer Susceptibility in Wild Animals

Wildlife cancer susceptibility varies markedly among species, reflecting differences in life-history traits, evolutionary histories, and species-specific cancer defense mechanisms [44]. A central concept underlying these interspecific differences is Peto’s paradox, which describes the lack of a positive relationship between cancer incidence and either body size or lifespan across species [45,46]. In principle, larger and longer-lived organisms should face a greater risk of malignant transformation. Such organisms possess more cells and undergo a greater number of cell divisions throughout life [47,48]. However, empirical observations indicate that cancer prevalence does not increase proportionally with body size or longevity, suggesting that natural selection has repeatedly favored the evolution of enhanced cancer-suppression mechanisms in species exposed to elevated oncogenic risks [49,50].

**Figure 3:**
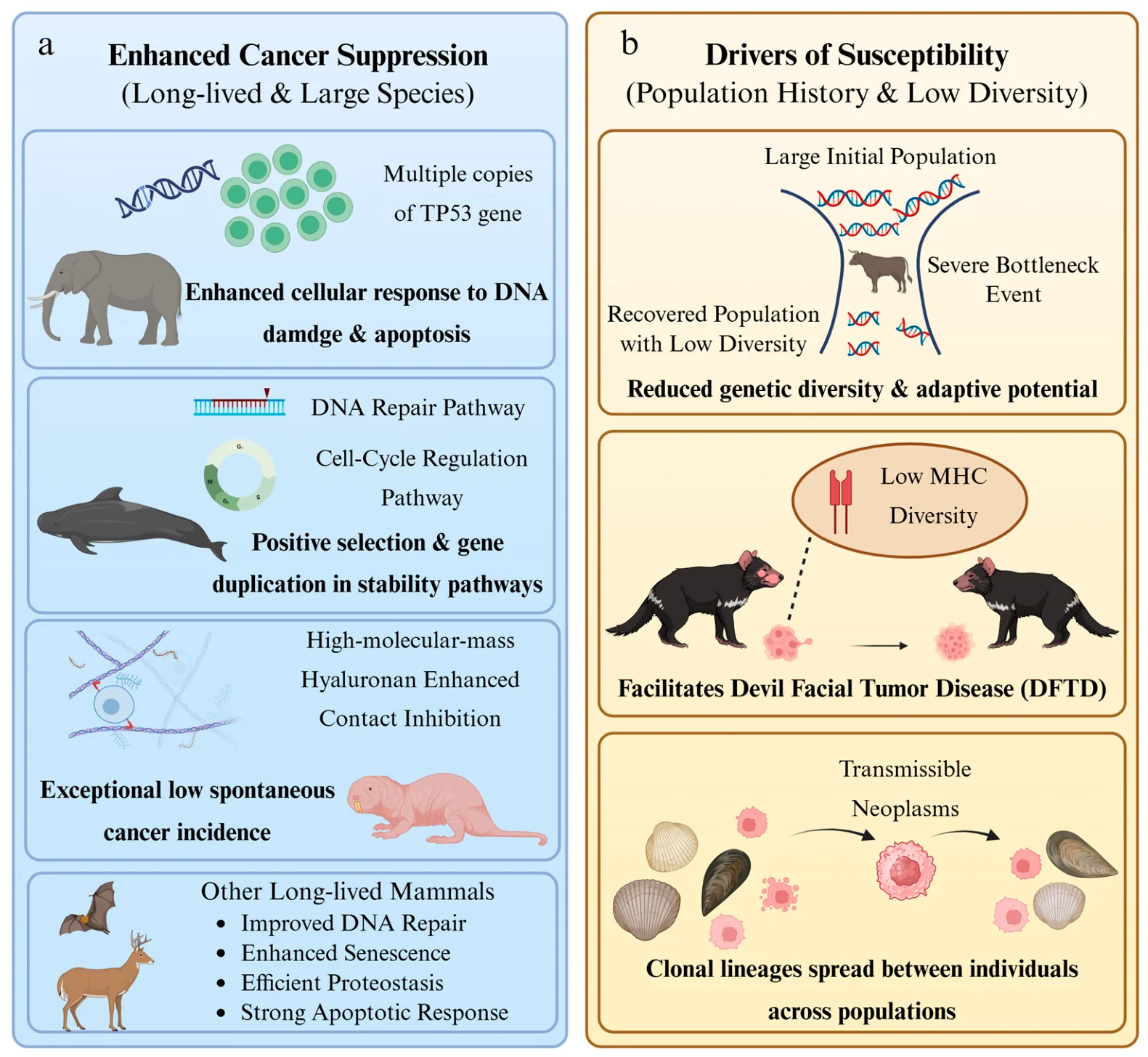
Intrinsic mechanisms and evolutionary drivers governing cancer susceptibility and resistance in wild animals. (**a**) Mechanisms of enhanced cancer suppression in long-lived and large-bodied species. (**b**) Evolutionary and ecological drivers increasing cancer susceptibility.

Evidence from comparative oncology demonstrates that multiple wildlife lineages have independently evolved mechanisms that mitigate cancer risk [51]. Elephants represent one of the best-characterized examples. Compared with humans, elephants possess multiple copies of the tumor suppressor gene TP53, which enhances cellular responses to DNA damage and promotes apoptosis of potentially malignant cells [52]. This tumor suppression mechanism is supported by field-derived wildlife evidence from genomic studies of both captive and wild elephant populations. These adaptations are thought to compensate for the increased cancer risk associated with large body size and extended lifespan [53]. Similarly, cetaceans exhibit remarkable longevity and body mass while maintaining relatively low cancer prevalence. Comparative genomic analyses have identified signatures of positive selection and gene duplication in pathways involved in DNA repair, cell-cycle regulation, and genome maintenance. These findings suggest that enhanced genomic stability contributes to cancer resistance in these species [54]. Additional examples of evolved cancer resistance have been documented in other long-lived mammals. The naked mole rat exhibits exceptionally low spontaneous cancer incidence and possesses unique biological characteristics that suppress tumor development, including the production of high-molecular-mass hyaluronan and enhanced contact inhibition mechanisms [55]. The anti-tumor function of hyaluronan is based largely on extrapolated inference from laboratory cell-culture assays, with limited validation across wildlife populations. More broadly, long-lived mammals often display improved DNA repair capacity, enhanced cellular senescence, efficient proteostasis, and stronger apoptotic responses, highlighting the diversity of evolutionary solutions to cancer suppression [56] (Figure 3a). Beyond genomic alterations and canonical tumor suppressor mechanisms, cancer susceptibility is also shaped by conserved regulatory networks involving signaling proteins, membrane transporters, and protein interaction systems. These molecular networks regulate fundamental cancer-related processes, including cellular proliferation, apoptosis, metabolic adaptation, stress responses, and immune regulation [57]. Membrane transporters, including ATP-binding cassette proteins such as ABCC4, influence cellular homeostasis by controlling the transport of metabolites, signaling molecules, and xenobiotic compounds, thereby affecting tumor cell survival and adaptation [58]. Although direct evidence from wildlife species remains limited, comparative analyses of conserved regulatory networks may reveal how different taxa evolve distinct mechanisms of cancer resistance and susceptibility.

Although these examples provide evidence that evolutionary adaptations can reduce cancer risk, current evidence does not support interpreting Peto’s paradox as a universal principle across vertebrate taxa [46]. Support for the paradox appears to depend on study design, taxonomic coverage, and the quality of available cancer datasets [59]. Much of the supporting evidence has been derived from mammals, particularly species maintained under long-term pathological surveillance in zoological collections, where cancer diagnosis is relatively standardized [60]. In contrast, comparative analyses across diverse terrestrial vertebrates often rely on heterogeneous datasets with uneven taxonomic representation, inconsistent diagnostic criteria, and limited pathological confirmation. These methodological differences may contribute to the conflicting conclusions reported across studies. Species-specific life-history traits, ecological adaptations, and cancer-suppression mechanisms may further contribute to interspecific variation in cancer susceptibility [44]. Therefore, Peto’s paradox should be regarded as an important but still debated evolutionary hypothesis rather than a universally validated biological rule [46]. Future studies integrating standardized wildlife surveillance, comparative pathology, and phylogenetically informed analyses will be essential for resolving these inconsistencies [61].

Despite the evolution of effective cancer-defense mechanisms in some taxa, substantial variation in susceptibility persists across wildlife species [43]. Differences in genetic architecture, immune surveillance, and population history can influence susceptibility to tumor development. Species that have experienced severe population bottlenecks may be particularly susceptible because reduced genetic diversity limits adaptive potential and weakens immune recognition of abnormal cells [62]. The Tasmanian devil provides a notable example, as low major histocompatibility complex (MHC) diversity facilitates the transmission of devil facial tumor disease (DFTD), one of the few naturally occurring transmissible cancers identified in vertebrates [63]. Long-term field studies have demonstrated that DFTD has caused severe population declines in Tasmanian devils across affected regions of Tasmania [64]. The disease reduces adult survival and alters population structure, illustrating how limited genetic diversity and impaired immune recognition can increase susceptibility to transmissible cancers [65]. Conservation strategies, including captive insurance populations and genetic management, aim to preserve adaptive potential and mitigate further population decline. Similarly, transmissible neoplasms have been reported in several marine bivalve species. In these systems, malignant cells can spread between individuals and establish clonal lineages that persist across populations, demonstrating how host genetic characteristics can shape cancer emergence and transmission dynamics [66,67,68]. These examples emphasize that cancer susceptibility is not determined solely by mutation accumulation but also by evolutionary processes affecting immune recognition, genetic diversity, and host–pathogen interactions [69] (Figure 3b). Collectively, evidence from comparative oncology indicates that wildlife cancer susceptibility is strongly influenced by intrinsic evolutionary mechanisms [9]. A balanced evaluation of the conflicting evidence surrounding Peto’s paradox further highlights the need to incorporate evidence quality into cross-species comparisons. The apparent resolution of Peto’s paradox through diverse cancer-defense adaptations highlights the role of natural selection in shaping species-specific resistance strategies [53]. At the same time, variation in genetic diversity, immune function, and evolutionary history contributes to marked differences in susceptibility among taxa [70]. Understanding these intrinsic determinants is essential for explaining patterns of cancer occurrence across wildlife species and for advancing the broader field of evolutionary and comparative oncology [8].

## 6. Methodological and Conceptual Barriers in Wildlife Cancer Research

Despite growing recognition of wildlife cancer as an emerging concern in biodiversity conservation and One Health, progress in understanding its ecological and evolutionary significance remains constrained by substantial methodological and conceptual barriers. Compared with infectious diseases, wildlife cancer presents unique challenges for wildlife surveillance because tumor development is typically chronic, multifactorial, and often asymptomatic during its early stages. Consequently, wildlife cancer in free-ranging populations is often detected opportunistically. This practice can substantially underestimate disease prevalence and limit our understanding of cancer distribution across species and ecosystems [5,7].

**Figure 4:**
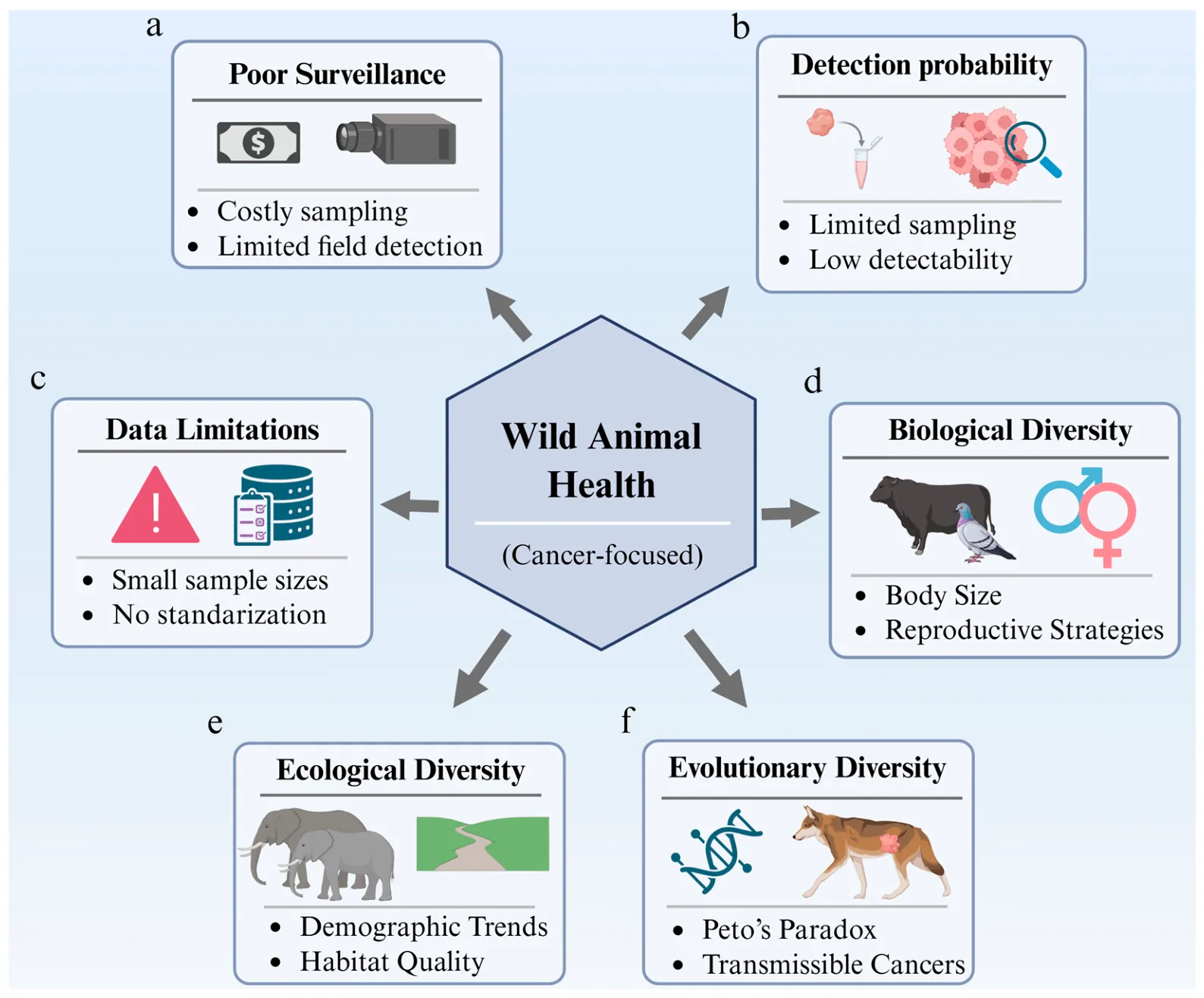
Methodological and conceptual barriers constraining wildlife cancer research within the wild animal health framework. (**a**) Poor surveillance owing to costly field sampling and logistical difficulties in remote environments. (**b**) Low detection probability resulting from limited sampling techniques, incidental detection, and low sensitivity of current diagnostic approaches under field conditions. (**c**) Data limitations characterized by small sample sizes, fragmented individual case reports, and a lack of standardized diagnostic criteria. (**d**) Biological diversity constraints in wildlife. (**e**) Ecological diversity gaps in wildlife. (**f**) Evolutionary diversity misconnections in wildlife.

One of the most important obstacles is the limited capacity for effective field surveillance and early diagnosis. Most wildlife species inhabit remote or inaccessible environments where repeated sampling is logistically difficult and financially expensive [71]. Routine clinical examinations are rarely feasible in free-ranging populations, while invasive sampling is often impractical because of ethical, conservation, and welfare considerations [72]. Consequently, current surveillance relies heavily on carcass examinations, incidental observations, or samples collected during unrelated ecological investigations, introducing substantial sampling bias and reducing the probability of detecting early-stage neoplasms [73] (Figure 4a). Although non-invasive approaches, including environmental DNA, circulating tumor DNA, molecular biomarkers, and imaging technologies, have shown considerable promise, their application remains constrained by insufficient cross-species validation, low sensitivity under field conditions, and the lack of standardized sampling protocols [74,75]. Technological constraints in long-term field monitoring, including limited tracking duration, restricted remote sensing capacity, and challenges in continuous environmental sampling, further reduce surveillance efficiency, particularly for highly mobile or cryptic species [76] (Figure 4a,b).

Methodological limitations inevitably lead to fragmented datasets, which currently represent one of the greatest barriers to wildlife cancer research [77]. Existing knowledge is largely derived from isolated case reports, opportunistic pathological investigations, and studies focusing on a limited number of charismatic or economically important species. Consequently, the available literature exhibits substantial taxonomic, geographic, and ecological biases [78,79]. Cancer occurrence remains poorly documented in many amphibian, reptilian, invertebrate, and small mammal species, making it difficult to determine whether apparent differences in cancer prevalence reflect genuine biological variation or simply uneven research effort [80]. These limitations are compounded by the lack of standardized diagnostic criteria and harmonized reporting systems. Variations in histopathological classification, terminology, sample preservation methods, and metadata collection among institutions and countries severely limit the comparability of datasets generated by different wildlife surveillance programs [81]. Unlike human oncology, where standardized cancer registries support long-term epidemiological analyses, wildlife cancer research lacks globally coordinated databases capable of facilitating comparative analyses across species and ecosystems [82]. Consequently, current estimates of wildlife cancer prevalence, incidence, and associated risk factors remain subject to considerable uncertainty [19] (Figure 4c).

Beyond methodological constraints, the remarkable biological diversity of wildlife introduces additional conceptual challenges for comparative analyses. Species differ substantially in lifespan, body size, reproductive strategies, immune function, and evolutionary history, all of which shape cancer susceptibility and tumor progression [49]. These intrinsic differences make it difficult to establish universal models describing cancer development across taxa. Consequently, findings obtained from well-studied mammals cannot always be extrapolated to birds, reptiles, amphibians, fishes, or invertebrates, highlighting the need for species-specific interpretations within broader comparative oncology frameworks [83] (Figure 4d). Moreover, cancer is increasingly recognized as an ecological and evolutionary process rather than merely an individual pathological condition [84]. Although substantial progress has been made in understanding transmissible cancers, Peto’s paradox, and species-specific cancer resistance mechanisms, these concepts remain insufficiently integrated into wildlife conservation biology [61]. Most conservation programs continue to assess population viability primarily through demographic trends, habitat quality, and infectious disease risks, whereas tumor susceptibility, evolutionary responses to carcinogenic environments, and long-term cancer dynamics are rarely incorporated into conservation assessments. Consequently, our ability to predict the effects of chronic environmental change on wildlife populations through cancer-mediated pathways remains limited [43] (Figure 4f).

These methodological and conceptual limitations also restrict the application of emerging analytical approaches that have transformed cancer research in humans and domestic animals [85]. Recent advances in artificial intelligence, machine learning, spatial epidemiology, and multi-omics integration have greatly improved cancer prediction, diagnosis, and risk assessment in clinical oncology [86]. However, applying these technologies to wildlife systems remains challenging. Available datasets are often characterized by small sample sizes, incomplete metadata, irregular temporal coverage, and substantial taxonomic heterogeneity [87]. Consequently, predictive models developed for human medicine are often difficult to transfer directly to wildlife populations without extensive ecological adaptation and rigorous validation [88]. In addition, the field deployment of advanced technologies in wildlife cancer research is constrained by logistical, infrastructural, and financial limitations. Unlike clinical settings, wildlife surveillance often requires sampling across remote and heterogeneous environments, where repeated capture, sample collection, and physiological monitoring are difficult to perform [71]. Moreover, multi-omics approaches require stringent sample preservation, specialized laboratory facilities, and complex bioinformatic pipelines, which are often unavailable in field-based conservation programs. AI-driven diagnostic models similarly require large, accurately annotated datasets, whereas wildlife studies often lack sufficient standardized pathological images and molecular profiles for robust training and validation [89]. The costs of long-term monitoring, cross-species data generation, and independent model validation further restrict large-scale implementation. Therefore, successful application of AI and multi-omics approaches will require not only technological innovation but also investment in field infrastructure, standardized protocols, and collaborative international surveillance networks [5].

The increasing complexity of anthropogenic environmental change further complicates wildlife cancer research [43]. Rather than being exposed to a single carcinogenic factor, wild animals are typically subjected to multiple environmental stressors, including chemical pollutants, habitat degradation, climate change, nutritional imbalance, and pathogen infection [90]. These interacting pressures may produce synergistic or cumulative effects on carcinogenesis, yet most current studies continue to investigate individual drivers independently [91]. As a result, the ecological mechanisms linking environmental change to cancer emergence remain insufficiently understood, limiting the development of reliable risk prediction models and effective conservation interventions [11] (Figure 4e). Another important limitation is the insufficient integration of cancer biology with ecology and conservation sciences. Compared with infectious diseases, wildlife cancer has only recently been recognized as a population-level conservation concern, and therefore remains largely absent from routine wildlife health assessments, biodiversity monitoring programs, and conservation planning [92]. Existing management strategies continue to prioritize habitat loss, invasive species, climate change, and infectious diseases, whereas chronic neoplastic diseases are rarely incorporated into conservation decision-making despite growing evidence that cancer can reduce individual fitness, reproductive success, and ultimately population viability in susceptible species [43].

However, the establishment of such international registries requires clearly defined organizational structures rather than data aggregation alone. A feasible framework should include a central coordinating body responsible for database governance, ethical oversight, and long-term maintenance [93]. Regional nodes could be operated by wildlife research institutions, veterinary organizations, conservation agencies, and governmental monitoring programs. Standardized data submission protocols should define minimum requirements for pathological classification, molecular information, environmental exposure records, geographic coordinates, and species-specific metadata. In addition, independent expert committees should be established to evaluate data quality, update diagnostic standards, and coordinate cross-regional validation. Sustainable operation will require long-term funding mechanisms, open-access policies with appropriate data governance, and international collaboration agreements to ensure continuous participation from diverse stakeholders. Such an organizational model would transform wildlife cancer databases from passive repositories into coordinated surveillance infrastructures. These systems could support comparative research, early risk detection, and conservation decision-making. Addressing these barriers will require continued improvements in surveillance methodology, data quality, and conceptual integration. Priority methodological needs include standardized diagnostic criteria, harmonized metadata collection, improved interoperability among wildlife cancer datasets, and stronger connections between ecological observations and molecular pathology. Progress in these areas will reduce current sources of uncertainty and provide a more reliable evidence base for comparative analyses across taxa. Collectively, overcoming these methodological and conceptual barriers represents a prerequisite for the implementation of interdisciplinary research frameworks discussed below.

## 7. Interdisciplinary Strategies for Wildlife Cancer Research and Conservation

Wildlife cancer research remains fragmented, and important methodological and conceptual barriers persist. Addressing these challenges requires a shift from descriptive approaches toward an integrated and operational framework grounded in the One Health paradigm. At the core of this framework is the establishment of a standardized and interoperable data infrastructure for wildlife cancer research. Current surveillance efforts are hindered by inconsistent reporting formats, heterogeneous diagnostic criteria, and the absence of centralized repositories capable of integrating pathological, ecological, and environmental datasets [82]. Therefore, the development of globally harmonized wildlife cancer registries, guided by the data principles, is essential to ensure data findability, accessibility, interoperability, and reusability [93]. Such platforms should integrate multi-source information, including histopathological records, molecular diagnostics, ecological metadata, and environmental exposure profiles, thereby enabling large-scale comparative analyses across species and ecosystems.

Building upon this data foundation, the second component of the framework involves the integration of advanced analytical and diagnostic technologies. Recent advances in artificial intelligence, machine learning, and multi-omics integration provide promising directions for improving cancer detection, classification, and risk prediction in wildlife populations. In particular, AI-assisted image analysis of histopathological samples, environmental DNA-based monitoring, and circulating tumor DNA detection represent promising tools for non-invasive and scalable surveillance [94]. Most AI surveillance systems have only undergone preliminary validation in small-scale pilot studies and have not yet been comprehensively validated across diverse wildlife taxa. However, to be effective in wildlife systems, these technologies must be adapted to low-data environments and validated across diverse taxa to account for evolutionary and ecological heterogeneity [95]. Representative interdisciplinary implementation tools, their current wildlife applications, major operational advantages, existing limitations, and validation requirements are summarized in Table 1.

Despite their considerable potential, the practical application of artificial intelligence and multi-omics approaches in wildlife cancer research remains constrained by several important limitations. Most available studies rely on relatively small sample sizes because tumor specimens from free-ranging wildlife are difficult to obtain. Incomplete clinical, ecological, and environmental metadata further restrict the integration and interpretation of multi-omics datasets [5,96]. In addition, substantial differences in genome quality, physiological characteristics, and evolutionary history among species complicate cross-species comparisons and reduce the transferability of computational models. Current artificial intelligence algorithms also lack sufficient external validation across diverse wildlife taxa, limiting their generalizability under field conditions. Therefore, current applications of artificial intelligence and multi-omics approaches remain primarily exploratory, requiring careful adaptation to wildlife-specific biological and ecological contexts.

**Table 1:** Representative interdisciplinary implementation tools for wildlife cancer research, together with their current applications, operational advantages, existing limitations, and validation requirements.

Domain	Key Approach	Implementation Requirements	Advantages	Limitations	Validation Requirements	Refs.
Molecular diagnostics	eDNA; PCR/qPCR; molecular profiling	Chelonia mydas: eDNA monitoring of ChHV5; Lepidochelys kempii: qPCR detection of ChHV5 in tumors	Non-invasive; sensitive; repeatable	Low target abundance; contamination; limited species-specific markers	Standardized sampling; cross-population validation	[97,98]
AI-based pathology	Digital histology; machine learning	Fish and bivalves: digital pathology for histological assessment	Rapid; reproducible; less observer variation	Limited datasets; taxonomic bias; limited wildlife validation	Multi-species datasets; independent validation	[99]
Eco-oncological modeling	Comparative oncology; spatial modeling	Parophrys vetulus: hepatic neoplasms and sediment contamination; Chelonia mydas: spatial analysis of fibropapillomatosis risk	Integrates cancer, ecology, and environment	Limited longitudinal data; causal uncertainty	Multi-site longitudinal validation	[19,100,101]
Environmental exposure analysis	Contaminant monitoring; tissue biomarkers	Zalophus californianus: carcinoma and environmental factors; Delphinapterus leucas: cancer in a contaminated environment	Identifies potential carcinogenic exposures	Complex exposure mixtures; uncertain causality	Long-term exposure–response studies	[102,103]
One Health surveillance	Integrated wildlife health monitoring	Sarcophilus harrisii: tumor-specific diagnosis of facial tumor disease; sea turtles: fibropapillomatosis monitoring	Integrates disease, population, and environmental data	Fragmented surveillance; heterogeneous protocols	Standardized reporting; longitudinal monitoring	[104]

The third component focuses on the development of coordinated governance structures within a One Health framework. Wildlife cancer surveillance should be explicitly integrated into existing global animal health and biodiversity monitoring systems, including those operated by veterinary, ecological, and public health organizations [105]. Strengthening institutional coordination among research institutions, conservation agencies, and international organizations can improve information exchange and promote coordinated wildlife cancer surveillance. In this context, wildlife cancer should be recognized as a component of broader environmental health surveillance, alongside infectious disease monitoring, pollution assessment, and ecosystem health indicators [106]. However, relevant cross-sector governance frameworks remain largely theoretical, with limited large-scale practical implementation across wild animal populations.

Finally, this framework must translate scientific insights into conservation-oriented decision-making. This requires the incorporation of cancer risk assessment into biodiversity conservation planning, particularly for endangered or small populations in which even modest reductions in individual fitness may have significant demographic consequences. Integrating cancer vulnerability indices into species risk assessments, together with genetic, ecological, and environmental parameters, may provide a more holistic evaluation of extinction risk. Moreover, linking environmental remediation strategies with habitat restoration and population management could help reduce carcinogenic exposure at the ecosystem level, thereby mitigating long-term disease burden in vulnerable wildlife populations [107]. However, standardized cancer vulnerability indices have not yet been validated across most wildlife taxa and their practical application still requires targeted field verification. Collectively, this multi-layered implementation framework emphasizes the need to transition from fragmented and reactive approaches toward a proactive, predictive, and integrative system of wildlife cancer management. By connecting data infrastructure, analytical innovation, governance coordination, and conservation action, the One Health paradigm can be operationalized in a manner that not only advances scientific understanding but also directly contributes to biodiversity conservation in an era of accelerating environmental change.

## 8. Future Priorities for Wildlife Cancer Research

**Figure 5:**
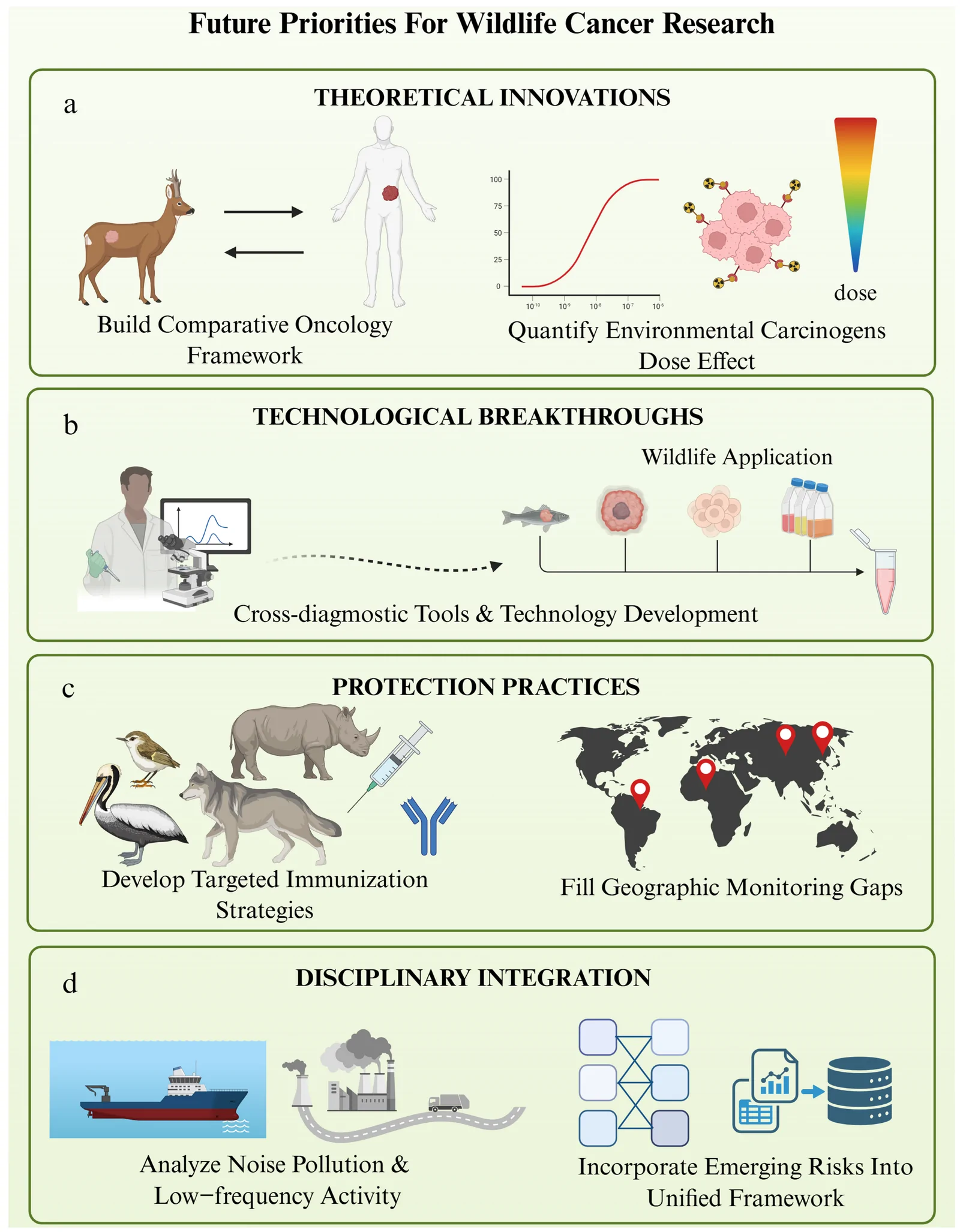
Future strategic priorities and implementation frameworks for wildlife cancer research across multiple scales. (**a**) Theoretical innovations focusing on building a comparative oncology framework to bridge human and animal research. (**b**) Technological breakthroughs driven by the development of cross-diagnostic tools and their direct clinical and surveillance applications in wildlife populations. (**c**) Conservation and protection practices aimed at developing targeted immunization strategies for cancer-susceptible populations. (**d**) Disciplinary integration leverages assessments of emerging anthropogenic stressors to strengthen coordinated wildlife cancer management under the One Health framework.

Addressing these challenges requires the identification of priority areas across multiple scales. The conservation significance of wildlife cancer should be evaluated according to its consequences for population viability rather than disease occurrence alone. Although no universally accepted prevalence threshold currently exists for wildlife cancers, conservation concern should increase when neoplastic diseases reduce survival, reproductive success, or recruitment, particularly in small or declining populations [108]. Cancer-related mortality may have disproportionate effects on vulnerable populations by increasing demographic stochasticity, altering age structures, and reducing adaptive potential. This risk may be particularly pronounced in species with restricted geographic distributions, low genetic diversity, slow life histories, or chronic exposure to environmental carcinogens. Such species may have limited capacity to compensate for additional mortality. Population-level impacts have been documented in several wildlife systems, including Tasmanian devil facial tumour disease and sea turtle fibropapillomatosis, in which severe disease can reduce individual fitness and, in some populations, contribute to demographic decline through increased mortality, impaired reproduction, or impaired reproduction [109]. Therefore, conservation assessments should integrate cancer prevalence with disease severity, demographic trends, ecological characteristics, and species-specific vulnerability rather than relying on fixed prevalence thresholds. Future technological development should prioritize cross-diagnostic tools for wildlife cancer detection and surveillance [107] (Figure 5b). Theoretical advances should strengthen comparative oncology frameworks and improve the quantification of dose–response relationships for environmental carcinogens [14]. They should also clarify the interactions among human, animal, and environmental health within the One Health framework (Figure 5a). Protection practices need to develop targeted immunization strategies for cancer populations [107]. Such vaccination schemes are only applicable to tumor lesions driven by persistent pathogens or naturally transmissible wildlife cancers rather than sporadic tumors induced by chemical pollutants or spontaneous genomic mutations [110]. Representative efforts include immunization strategies targeting Tasmanian devil facial tumour disease, which aim to induce anti-tumour immunity against transmissible cancer cells [111]. Immunotherapies for non-infectious sporadic wildlife cancers remain limited by undefined tumor-specific antigens and complex interspecific immune variation. No mature injectable vaccine protocols have been validated for these disease types, and at the same time, to fill the gap in geographic monitoring (Figure 5c). Interdisciplinary integration should be guided by the One Health framework, emphasizing interconnected biological, ecological, and environmental networks [112]. This approach should also incorporate emerging risks, including the mechanisms underlying noise pollution and disturbances caused by low-frequency anthropogenic activities, into unified analytical frameworks [113] (Figure 5d).

Collectively, these proposed frameworks should be regarded as long-term research directions rather than immediately applicable conservation tools. Their successful implementation will depend on continued methodological development, interdisciplinary collaboration, and robust validation across diverse wildlife populations and ecological settings.

## 9. Conclusions

**Figure 6:**
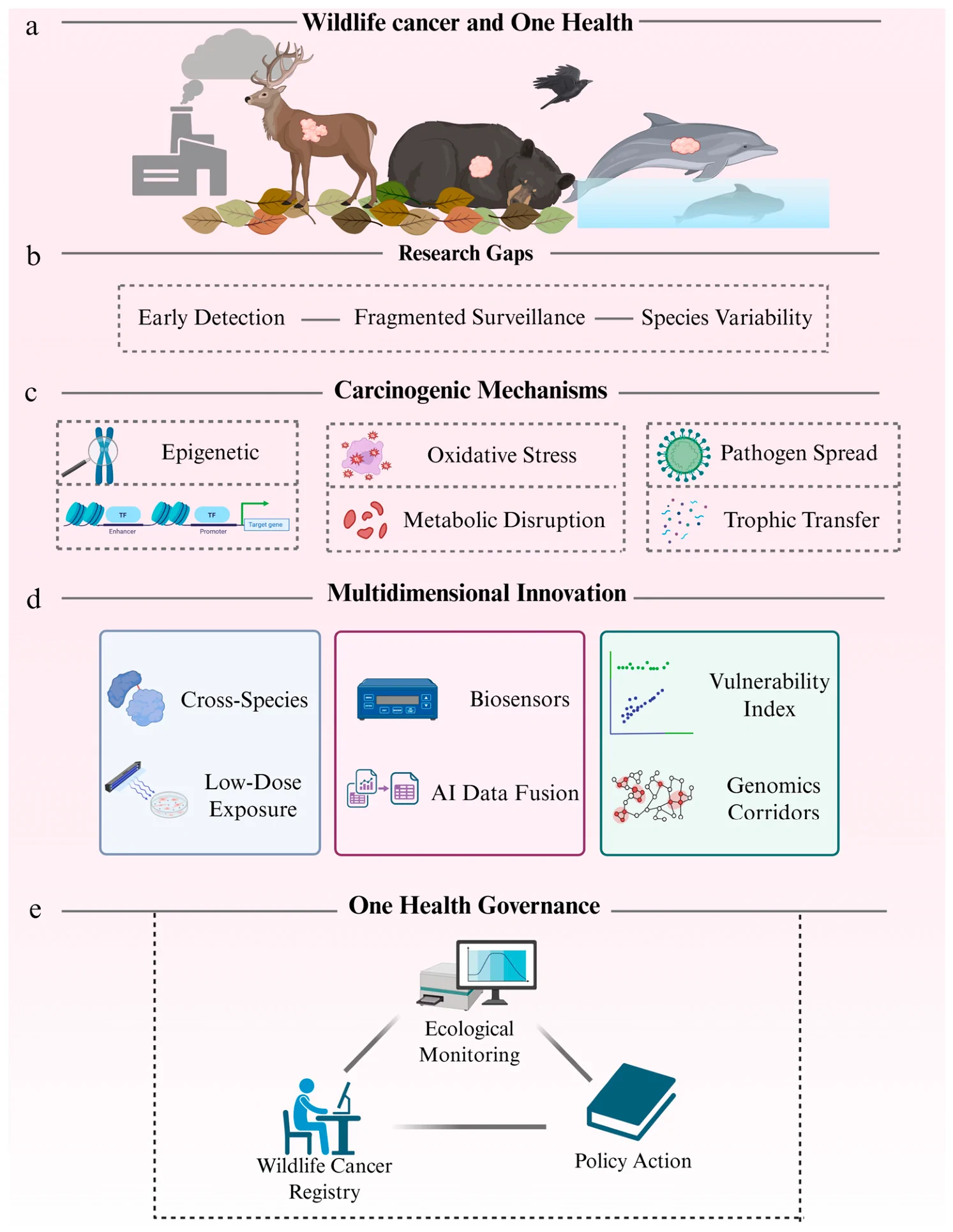
Comprehensive summary framework and future outlook for wildlife cancer research and governance. (**a**,**b**) Primary wildlife cancer research gaps within the One Health framework. (**c**) Multi-pathway carcinogenic mechanisms and ecological amplification effects. (**d**) Multidimensional innovations required across experimental models. (**e**) A coordinated One Health governance matrix integrating real-time ecological monitoring, a global wildlife cancer registry, and active policy interventions.

In conclusion, wildlife cancer, an integral component of the One Health framework, remains constrained by insufficient early detection, fragmented surveillance networks, and substantial interspecific variation in cancer susceptibility and resistance (Figure 6a,b). Environmental carcinogens contribute to tumorigenesis through interconnected pathways, including epigenetic dysregulation, oxidative stress, metabolic dysfunction, pathogen-mediated oncogenic processes, and trophic contaminant transfer (Figure 6c). Addressing these barriers demands cross-field innovations spanning comparative oncology, long-term ecological monitoring, biosensing tools, AI-powered data fusion and unified surveillance protocols, alongside coordinated governance guided by the One Health paradigm (Figure 6d,e). For conservation institutions, priorities should include integrating cancer surveillance into routine wildlife health monitoring, standardizing diagnostic and metadata protocols, and strengthening data sharing across veterinary, ecological, and conservation sectors. It should be noted that several conceptual schematic diagrams in this review are constructed to generalize shared mechanisms and research paradigms from a body of disparate primary studies. Due to considerable heterogeneity in experimental designs, animal/cell models, measurement endpoints, and data processing methods among individual reports, it is impractical to integrate standardized, comparable quantitative metrics into these visual frameworks. We clarify this limitation to avoid overinterpretation of the schematic illustrations. Overall, this review integrates ecological, evolutionary, molecular, and conservation perspectives to clarify the drivers and consequences of wildlife cancer and provides a One Health framework for strengthening wildlife health surveillance, biodiversity conservation, and environmental hazard assessment.

## Data Availability

No new data were created or analyzed in this study. Data sharing is not applicable to this article.
